# Acute Mastoiditis With Intracranial Complications in a Young Adult With History of Cranioplasty: The Rule in the Post-Antibiotic Era

**DOI:** 10.7759/cureus.9452

**Published:** 2020-07-29

**Authors:** Anthony J Febres Aldana, Paola Rios

**Affiliations:** 1 Internal Medicine, Mount Sinai Medical Center, Miami Beach, USA

**Keywords:** mastoiditis, epidural abscess, biofilm, methylmethacrylate, otitis media, latent mastoiditis

## Abstract

The presentation of acute mastoiditis has become erratic over the last decades secondary to the wide use of broad-spectrum antibiotics. While establishing this diagnosis requires a high degree of suspicion, imaging is necessary because of the concurrence of intracranial complications. Therefore, the diagnostic hypothesis of acute mastoiditis must prompt the evaluation for the presence of intracranial complications, such as intracranial epidural abscess (ICEA) formation. Hereby, we present a case of a 33-year-old woman presented to the ED of our institution with symptoms consistent with acute mastoiditis. She had a history of a methyl-methacrylate (MMA) cranioplasty performed 10 years before presentation for debulking of an epidermoid cyst. She was found to have the formation of an ICEA. Development of infection in patients with MMA cranioplasty is seen most commonly within one year of the implantation with the late presentation being a rare occurrence. Treatment in these cases is comprised of antibiotic therapy, but most importantly of the removal of the infected foreign material to prevent further complications from the infection.

## Introduction

Acute mastoiditis refers to the inflammation of the mastoid cells of the temporal bone. This phenomenon is most commonly seen in the setting of long-term bacterial colonization or because of bacterial mobilization from surrounding non-infected organs. With similar pathophysiology to the infection of other bones, it usually arises from a complicated middle ear inflammation and therefore its occurrence is most common in the pediatric population [1,2]. Due to its anatomy, mastoiditis can lead to the development of extra-axial intracranial collections, extension of the inflammation to the meninges, or venous sinus thrombosis, all these termed intracranial complications. In the pre-antibiotic era, the outcomes of the disease were catastrophic; therefore there is a need to recognize these complications promptly in order to decrease the morbidity and mortality of this ailment. Moreover, the presence of prosthetic hardware in the tissue increases the likelihood of these complications. Cranioplasty, done for both functional and cosmetic reasons in the presence of a calvarial defect, can complicate with infections, which may not present itself months following the surgery. Some materials are recognized to carry an increased risk for infection [3,4].

Hereby, we present the occurrence of mastoiditis in an adult female with the rapid development of an epidural abscess, 10 years after the removal of an epidermoid cyst done via a right retromastoid craniectomy with cranioplasty and implantation of a methyl-methacrylate (MMA) prosthesis. 

## Case presentation

**Figure 1:**
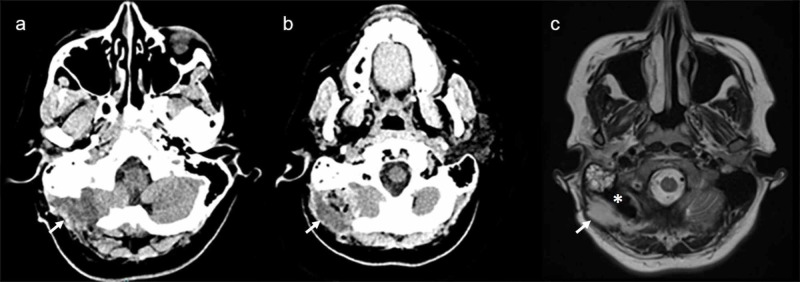
Brain imaging obtained before surgery (a and b) CT imaging of the mastoid area obtained upon presentation showing a large irregular thick-walled mass in the remnant cavity from the dermoid cyst removal 10 years before. (c) Fluid-attenuated inversion recovery (FLAIR) magnetic resonance imaging (MRI) sequence obtained before the first surgery which confirms the presence of a right occipital collection with central restricted diffusion (sequence not shown) and peripheral enhancement, consistent with an abscess formation (arrow) extending to the epidural space, and remnant cavity from the dermoid cyst removal in the past (star).

A 33-year-old female with a past medical history remarkable for a right retromastoid debulking of an epidermoid cyst via right retromastoid craniectomy with cranioplasty 10 years before presentation visited the emergency department of our institution complaining of a four-day history of right-sided ear ache, fever, and chills. On the second day of symptom onset, the patient was evaluated by her primary care physician, and diagnosed her with acute otitis media (AOM) and prescribed oral amoxicillin and neomycin with polymyxin eardrops. On the fourth day of her symptoms, she developed a severe pressure-like headache in the mastoid and occipital area, which persisted despite taking non-steroid anti-inflammatory agents. When she presented to the emergency department, her vital signs were remarkable for a heart rate of 108 beats per minute, a normal respiratory rate, and blood pressure of 108 over 76 mmHg. She was afebrile. Her physical exam revealed the presence of exquisite right mastoid tenderness. The otoscopic examination was limited due to cerumen present in the outer ear canal. There was no neck rigidity and her neurological examination was unremarkable. Her hematologic examination revealed a hemoglobin 12.8 g.dL^-1^ with a hematocrit of 38.9%, platelets were 1.99 x 10^5^ cells.µL^ -1^, and white blood cell count 19.06 x 10^3^ cells.µL^-1^ with neutrophil predominance of 83%. Her chemistry revealed normal electrolytes and normal kidney function. Blood glucose was 103 mg.dL^-1^, with a hemoglobin A1c of 5.1%. Liver function tests (LFTs) were unremarkable. Erythrocyte sedimentation rate (ESR) was mildly elevated at 22 mm.hr^-1^ with a normal C-reactive protein (CRP). CT imaging of the head showed a large thick-walled enhancing mass occupying the postoperative resection cavity involving the right occipital bone with extension to the epidural space and extensive right mastoiditis (Figure 1).

Magnetic resonance imaging (MRI) of the head was then performed, confirming the presence of a collection in the right occipital area, consistent with an epidural abscess, aside from opacification of the right mastoid system and a residual dermoid lesion in the prepontine cistern and clivus. The patient was admitted to the medical ward and a right posterior fossa craniectomy was performed for evacuation of the epidural abscess, which resulted in drainage of a purulent collection and removal of the MMA cranioplasty material. In light of overt purulent material, sampling of the bone was deferred. However, it appeared to be without evidence of osteomyelitis.

The patient was then initiated on empiric intravenous antibiotics with vancomycin, cefepime, and metronidazole. Cultures from blood and the drainage material showed no growth of organisms. Thirteen days after the first surgery, the patient had a right mastoidectomy with obliteration of the mastoid by placing abdominal fat in the mastoid cavity with a titanium mesh cranioplasty. On this second surgery, mastoid air cells infiltrated with MMA and bone wax were found, associated with an inflamed mastoid mucosa indicative of mastoiditis. Culture of this tissue showed no bacterial growth. Intravenous antibiotic regimen was switched to oral metronidazole, doxycycline, and levofloxacin. The patient was discharged from the hospital with instructions to continue the antibiotics for two more weeks.

## Discussion

The diagnosis of acute mastoiditis is based on the presence of post-auricular swelling, erythema, tenderness of the mastoid, protrusion of the auricle, and fever, in the presence or not of middle ear inflammation. However, in adults, it requires a high grade of suspicion as the clinical presentation is more frequently atypical [5]. Indeed, the classic signs are often absent and the patient presents many days after the development of the mastoid inflammation, by the time when complications have already established [6]. The development of intracranial complications is most commonly seen in patients with latent mastoiditis, exemplified by the clinical presentation of our patient [7]. Secondary to the widespread use of antibiotics over the recent decades, the incidence of mastoiditis has decreased, imposing an additional challenge in the interpretation of physical findings or diagnostic tests to identify this entity, with imaging thus being necessary. Interdisciplinary management has to be established quickly to decide the need for surgical intervention and for the choice of antibiotic therapy. The presence of foreign materials increases the risk of complicated mastoiditis. A history of trans-mastoid surgical procedures, the presence of an osseous defect that facilitates the communication between mastoid cells and intracranial epidural space, and the presence of prosthetic materials in the mastoid increase the risk of having a more complicated course of the disease [8].

Intracranial complications of acute mastoiditis are managed with mastoidectomy, which has been associated with an increased risk in recurrence of mastoiditis in pediatric cases. Groth et al. showed that in a period of 14 years, recurrence was 5% in patients with acute mastoiditis, were 75% had a prior mastoidectomy and 1% developed an intracranial complication. Authors speculated that the increase recurrence could be secondary to an easier access to the mastoid cavity [9]. Therefore, we need to consider it as a possibility in a patient who presents with new otalgia and even more if the patient had a prior history of cranioplasty plus mastoidectomy like in the present case.

Microbiology of mastoiditis varies according to the age group analyzed, with *Streptococcus pneumoniae* found in as much as one-third of the cases of pediatric cases, followed in frequency by S*treptococcus pyogenes*. In adults, the relationship inverts and *S. pyogenes *is most commonly seen [10]. Other pathogens such as *Pseudomonas aeruginosa*, *Corynebacterium diphteriae*, and *Escherichia coli* can be found as causative agents of mastoiditis in adults. In particular, those cases with intracranial complications are more often due to *S. pneumoniae* except for those cases complicated with an ICEA in the setting of a cranioplasty, where *Staphylococcus aureus* is the leading etiologic agent owing to its ability to produce biofilms. Biofilm-associated infections result in the induction of a bacterial phenotype characterized by slow bacterial growth, expression of genes that allow intercellular communication among bacteria, termed quorum-sensing system (QS), and the expression of antibiotic resistance genes [11]. The formation of a biofilm begins with the initial bacterial attachment to the surface, with subsequent formation of microcolonies and the maturation of the biofilm architecture. The result is the end dispersion of the biofilm which enables bacteria to move to a new infection site forming new biofilms [12]. The bacterial composition of biofilms can be heterogeneous making it possible that the infection in our patient was polymicrobial in origin, with different bacterial species each with different susceptibility profiles. It is unclear if this factor could influence the outcome as literature on the topic is limited, but contrary to what one may hypothesize, outcomes measures such as 30-day mortality seem to be independent of the number of bacterial species involved [13].

From a therapeutic standpoint, the presence of a biofilm surface translates in a more difficult to treat infection where removal of the prosthetic material is mainstay, with the addition of broad-spectrum antibiotic coverage against aerobic and anaerobic cocci and bacilli. The duration of antibiotic therapy for ICEAs is highly variable, with a recommendation of six through eight weeks, although the duration can be less if surgical management is optimally performed [6]. Surgical removal of the infected material is necessary because it functions as a diffusion barrier decreasing the antibiotic penetrance. MMA surfaces have a higher viscosity and thus a lower biocide agent penetration with lower rates of bacterial killing than those seen in other materials [14].

Several strategies to treat biofilm-associated infections can be hypothesized. These include avoidance of bacterial adhesion to susceptible surfaces, disruption of biofilm formation, and inhibition of the bacterial communication by interfering with the QS system [15]. Nitric oxide has been shown to be an exemplary molecule in this setting by improving bacterial clearance when coadministered with fluoroquinolones to biofilms grown in vitro that contain either gram-positive or gram-negative bacteria [12,16]. Similarly, the implementation of nanoparticles (NPs) in the elaboration of the prosthetic hardware can decrease the risk of biofilm formation by altering their biophysical properties, conferring catalytic properties. Copper oxide (CuO) has significant antimicrobial activity against Gram-negatives but a lesser antimicrobial effect on Gram-positives. Conversely, zinc oxide (ZnO) is better at the inhibition and killing of Gram-positive bacteria. The use of composite NPs, such as cetyl-trimethyl ammonium bromide (CTAB) capped CuO NPs is a promising way to inhibit both Gram-positive and Gram-negative bacterial growth [17].

## Conclusions

Acute mastoiditis is a rare infection that can affect the adult population with an atypical clinical presentation. The presence of a prior history of cranioplasty with MMA increases the likelihood of the formation of bacterial biofilms, which translates into a more insidious and complicated course of the disease, including the development of ICEAs. Clinicians need to be aware that late intracranial complications from mastoiditis in adults with a history of a cranioplasty can be seen many years later. Current research demonstrates the progress in developing materials that disrupt the growth of bacteria. However, research focused on the employment of these tools in the clinical setting is necessary.
